# Treatment Pattern and Outcomes with Systemic Therapy in Men with Metastatic Prostate Cancer in the Real-World Patients in the United States

**DOI:** 10.3390/cancers13194951

**Published:** 2021-09-30

**Authors:** Umang Swami, Jennifer Anne Sinnott, Benjamin Haaland, Nicolas Sayegh, Taylor Ryan McFarland, Nishita Tripathi, Benjamin L. Maughan, Nityam Rathi, Deepika Sirohi, Roberto Nussenzveig, Manish Kohli, Sumanta K. Pal, Neeraj Agarwal

**Affiliations:** 1 Division of Oncology, Department of Internal Medicine, Huntsman Cancer Institute, University of Utah, Salt Lake City, UT 84112, USA; Umang.Swami@hci.utah.edu (U.S.); jsinnott@stat.osu.edu (J.A.S.); ben.haaland@hci.utah.edu (B.H.); Nicolas.Sayegh@hci.utah.edu (N.S.); Taylor.McFarland@hci.utah.edu (T.R.M.); Nishita.Tripathi@hci.utah.edu (N.T.); Benjamin.Maughan@hci.utah.edu (B.L.M.); Nityam.Rathi@hci.utah.edu (N.R.); Roberto.Nussenzveig@hci.utah.edu (R.N.); manish.kohli@hci.utah.edu (M.K.); 2 Department of Internal Medicine, University of Utah, Salt Lake City, UT 84132, USA; 3 Department of Pediatrics, University of Utah, Salt Lake City, UT 84108, USA; 4 Department of Statistics, The Ohio State University, Columbus, OH 43210, USA; 5 Department of Population Health Sciences, University of Utah, Salt Lake City, UT 84108, USA; 6 Department of Pathology, University of Utah and ARUP Laboratories, Salt Lake City, UT 84112, USA; Deepika.Sirohi@hsc.utah.edu; 7 Department of Medical Oncology & Experimental Therapeutics, City of Hope Comprehensive Cancer Center, Duarte, CA 91010, USA; spal@coh.org

**Keywords:** abiraterone, enzalutamide, docetaxel, novel hormonal therapies, comparative effectiveness, real-world treatment pattern, metastatic prostate cancer

## Abstract

**Simple Summary:**

Novel hormonal therapies (such as abiraterone and enzalutamide) and docetaxel are approved treatments for metastatic prostate cancer. Upfront use of these agents has been shown to improve overall survival. However, we do not know the real-world treatment patterns of these agents or the comparative effectiveness of these agents after treatment with a prior novel hormonal therapy in patients with metastatic prostate cancer. In this large study, we found that most patients with metastatic prostate cancer received only androgen deprivation therapy as upfront therapy without novel hormonal therapies or docetaxel. In patients treated with one novel hormonal therapy, alternate novel hormonal therapy was the most common next therapy and was associated with improved overall survival over docetaxel with the caveat of this being a non-randomized comparison. The study’s limitations also include its retrospective design.

**Abstract:**

Background: Both novel hormonal therapies and docetaxel are approved for treatment of metastatic prostate cancer (mPC; in castration sensitive or refractory settings). Present knowledge gaps include lack of real-world data on treatment patterns in patients with newly diagnosed mPC, and comparative effectiveness of novel hormonal therapies (NHT) versus docetaxel after treatment with a prior NHT. Methods: Herein we extracted patient-level data from a large real-world database of patients with mPC in United States. Utilization of NHT or docetaxel for mPC and comparative effectiveness of an alternate NHT versus docetaxel after one prior NHT was evaluated. Comparative effectiveness was examined via Cox proportional hazards model with propensity score matching weights. Each patient’s propensity for treatment was modeled via random forest based on 22 factors potentially driving treatment selection. Results: The majority of patients (54%) received only androgen deprivation therapy for mPC. In patients treated with an NHT, alternate NHT was the most common next therapy and was associated with improved median overall survival over docetaxel (abiraterone followed by docetaxel vs. enzalutamide (8.7 vs. 15.6 months; adjusted hazards ratio; aHR 1.32; *p* = 0.009; and enzalutamide followed by docetaxel vs. abiraterone (9.7 vs. 13.2 months aHR 1.40; *p* = 0.009). Limitations of the study include retrospective design.

## 1. Introduction

Based on significant improvement in overall survival in randomized controlled trials, docetaxel and novel hormonal therapies (NHTs) such as abiraterone or enzalutamide, were first approved for metastatic castration-resistant prostate cancer (mCRPC), and subsequently for men with metastatic castration-sensitive prostate cancer (mCSPC) [1,2,3,4,5]. However, contemporary data of treatment patterns are lacking to demonstrate whether these successes are reaching the real-world patients with metastatic prostate cancer (mPC) in the United States. 

Another knowledge gap pertains to treatment selection in the real world after the progression of mPC on one NHT. In this setting, both NHTs and docetaxel are available and considered standard of care [6]. However, the efficacy of docetaxel and NHT after prior NHT use in mPC has not been compared in a randomized controlled trial, and/or in any large real-world patient-level dataset. In clinical trials, the time on alternate NHT after disease progression on one NHT has been 3.6 to 5.7 months [7,8]. Clinical trials have not evaluated the efficacy of docetaxel after one prior NHT but the median duration of docetaxel as the first subsequent therapy in the registration trial of abiraterone in chemotherapy-naïve mCRPC setting (COU-AA-302 trial) was 3.02 months (interquartile range 0.95–5.72) [9].

The current lack of real-world treatment patterns and treatment-related outcomes hamper efforts on improving patients’ access to these therapeutic agents as well as designing of clinical trials in these men. Furthermore, a lack of comparative effectiveness data prevents patients from making best-informed treatment decisions. Drug development is hindered, due to lack of information on estimates of progression-free survival and overall survival (OS) on subsequent therapies after treatment with one NHT, as well as due to challenges in terms of selection of the best control arm in the randomized trials in this setting. In this study, our objective was to fill in these knowledge gaps by evaluating the treatment patterns in patients with new mPC and comparing the efficacy of an alternate NHT (abiraterone or enzalutamide) versus docetaxel after prior therapy with only one NHT (abiraterone or enzalutamide) in real-world patients with mPC.

## 2. Materials and Methods

### 2.1. Study Population

De-identified patient-level data from patients with mPC were extracted from the Flatiron Health Electronic Health Record database. The Flatiron database consists of nationally representative real-world data from community practices and academic medical centers from 2011 through the present and contains structured and unstructured data curated via technology-enabled abstraction and supplemented with third-party death information. Details of the Flatiron database and its comparison with other real-world databases have been discussed elsewhere [10]. This study was approved by the Institutional Review Board at the University of Utah (IRB_00067518, last approved 9 July 2021). 

Eligibility criteria: patients with mPC diagnosed from January 2011 and treated through 30 September 2019; the patient had some evidence of contact within 180 days of diagnosis of metastatic disease to ensure that the patient was actively engaged in care at the data providing institution, and availability of treatment data after diagnosis of metastatic disease. For comparative effectiveness analysis, patients also needed to receive one NHT for mPC followed by an alternate NHT or docetaxel. Patients with systemic therapy with any anti-cancer drug (except ADT) prior to first NHT were excluded. For the purpose of comparative effectiveness analysis use of first NHT was considered first line (1L) therapy. Receipt of a second NHT or docetaxel after one prior NHT was considered second-line (2L) treatment. The follow-up period was until 9 September 2019. All patients were required to have begun 2L therapy at least 6 months before the end of the follow-up period. Patients were excluded if they received any other anti-cancer agent in 1L or 2L, or if they had prior exposure to NHT or docetaxel in the non-metastatic setting. 

### 2.2. Outcome Definitions for Comparative Effective Analysis

OS was defined as the time from the start of 2L therapy to death from any cause. Among men who did not die during follow-up, censoring time was defined as the time of most recent contact in the data, which could have been a therapy end date or a visit, drug episode, or medication order. Time to initiation of 3L therapy or death (TTTTD) was defined as the time from the start of 2L therapy to the start of third-line (3L) therapy, or death. Among men who did not initiate a 3L therapy and died, death within 180 days after the end of 2L therapy was considered an event. Patients who died after the 180-day window were censored at the time of the last contact since we would anticipate that many of these patients were pursuing a 3L treatment at a different institution. Among men not pursuing 3L who did not die, censoring time was defined as the time of most recent contact in the data.

### 2.3. Statistical Analysis

Receipt of systemic therapy at the time of diagnosis of metastatic prostate cancer, and subsequent therapies and treatment patterns from 2011 to 2019 were summarized descriptively. For a comparative effectiveness study, the analyses were performed separately among 1L abiraterone and 1L enzalutamide patients. Patients treated with 2L NHT and docetaxel were compared at baseline and pre-2L characteristics using Wilcoxon rank-sum tests for quantitative variables and chi-squared tests for categorical variables. The survival outcomes of interest (TTTTD and OS) were compared visually without any adjustment using Kaplan–Meier survival curve estimates, and median survival times were estimated overall and in groups defined by time on 1L therapy. To account for patient characteristics that may affect both treatment selection and outcomes, Cox proportional hazards models with propensity score weighting were used. The probability of receiving docetaxel vs. alternate NHT was estimated using a random forest approach [11], with candidate variables including: Gleason score at initial diagnosis; prostate-specific antigen (PSA) at the diagnosis of metastatic disease and at start of 1L (an indirect measure of disease volume); insurance status, which may influence the selection of 2L therapy (most recent reported payer prior to the 2L start date, including commercial health plan, Medicaid, Medicare, other government, other payer, and patient assistance program); age and year at the time of starting 2L; race; time on ADT-only therapy after metastasis; time on 1L therapy and whether the patient was considered hormone sensitive at time of 1L initiation; the number of diagnoses in the medical records; indicators for diagnosis codes for visceral metastasis, any other specific metastasis, diabetes, heart failure, or neuropathy; Eastern Cooperative Oncology Group (ECOG) performance status in the 3 months prior to 2L start; and PSA, lactate dehydrogenase (LDH), alkaline phosphatase, and hemoglobin in the 3 months prior to starting 2L therapy. In all cases, a separate category was coded for missing values. The propensity scores were used to calculate matching weights, targeting the same estimand as 1:1 matching of treatment groups on combinations of potential confounders [11]. Covariate balance was assessed via weighted tests and by examining standardized mean differences. The weights were then used in Cox proportional hazards models to evaluate the effect of docetaxel compared with other NHT, balanced on potential confounders.

In addition to the main analyses, subgroup analyses were performed based on age, Gleason score, time on 1L therapy, performance status, alkaline phosphatase, PSA, LDH, and hemoglobin at 2L initiation. Propensity scores were recalculated within each subgroup. Additionally, selected post-2L characteristics, including post-2L ECOG and numbers of post-2L therapies, were compared across the groups. All analyses were performed in R version 4.0.2, using packages ggplot2, randomForest, survey, survival, and tableone.

## 3. Results

**Figure 1:**
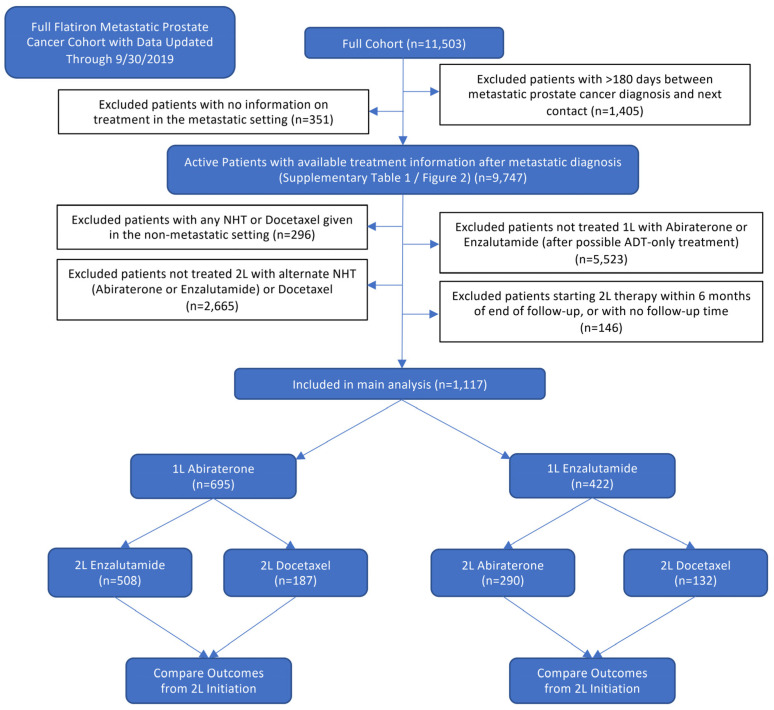
Flow diagram depicting stepwise patient selection.

A flow diagram illustrating the selection of patients for both the full cohort and 2L analyses is presented in Figure 1.

### 3.1. Treatment Patterns in Patients with Metastatic Prostate Cancer

**Figure 2:**
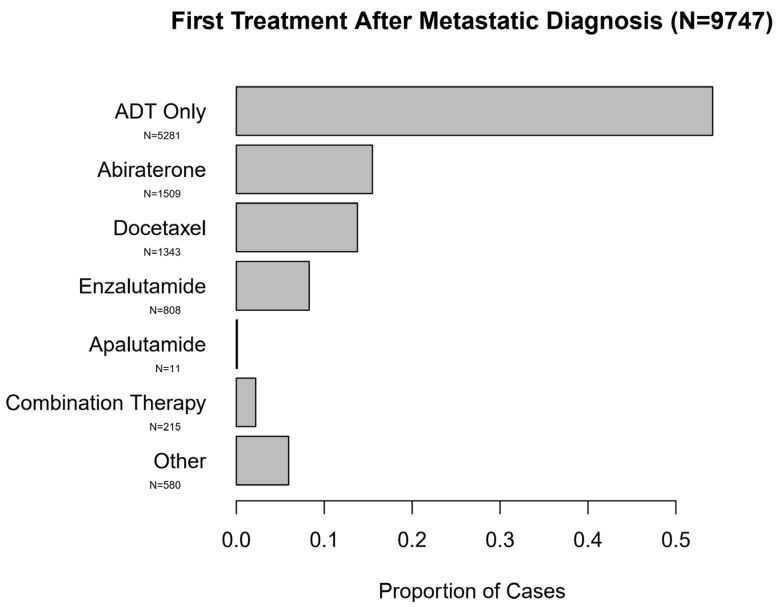
First Treatment After Metastatic Diagnosis (*N* = 9747).

**Figure 3:**
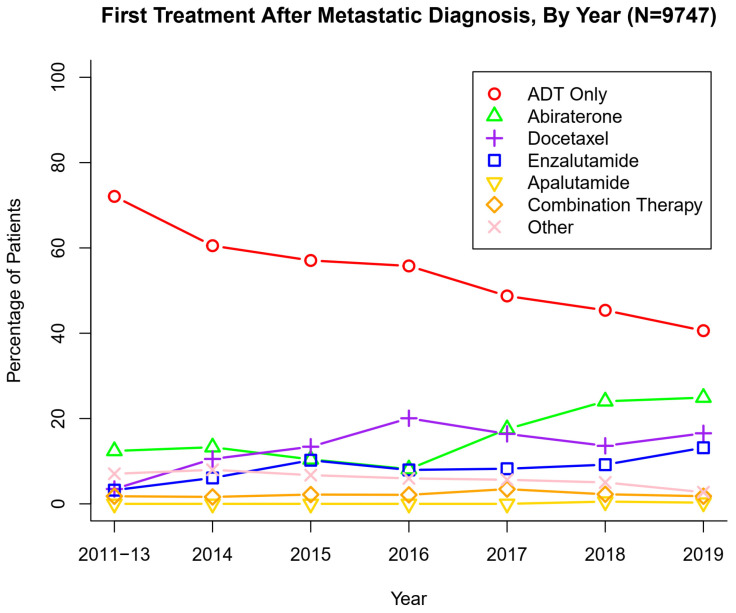
First Treatment After Metastatic Diagnosis, By Year (*N* = 9747).

Table S1 summarizes patient characteristics for the full cohort of mPC patients (*N* = 9747 after exclusions from initial *N* = 11,503) and Figure 2 summarizes treatment patterns after diagnosis of mPC. After the diagnosis of new mPC disease, 54.2% of patients were treated with ADT only. Abiraterone (15.5%) was the most frequently used intensifying agent, followed by docetaxel (13.8%) and enzalutamide (8.3%). The yearly trend of use of therapeutic agents for new diagnosis of mPC is presented in Figure 3 which demonstrates a gradual but encouraging increase in the use of NHTs at the time of onset of metastatic prostate cancer. 

**Figure 4:**
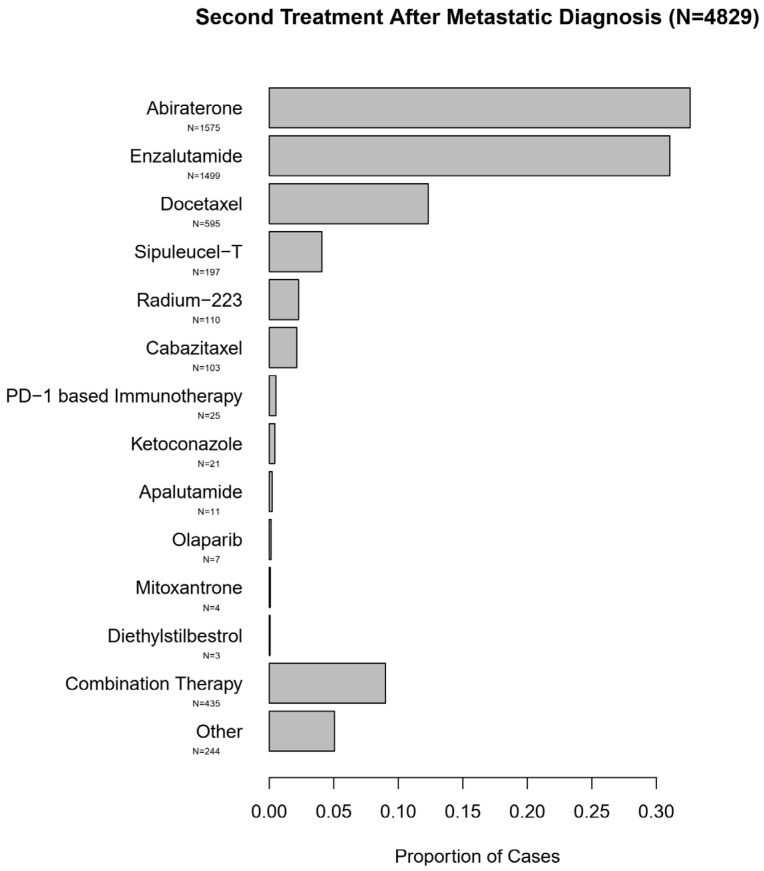
Second Treatment After Metastatic Diagnosis (*N* = 4829).

**Figure 5:**
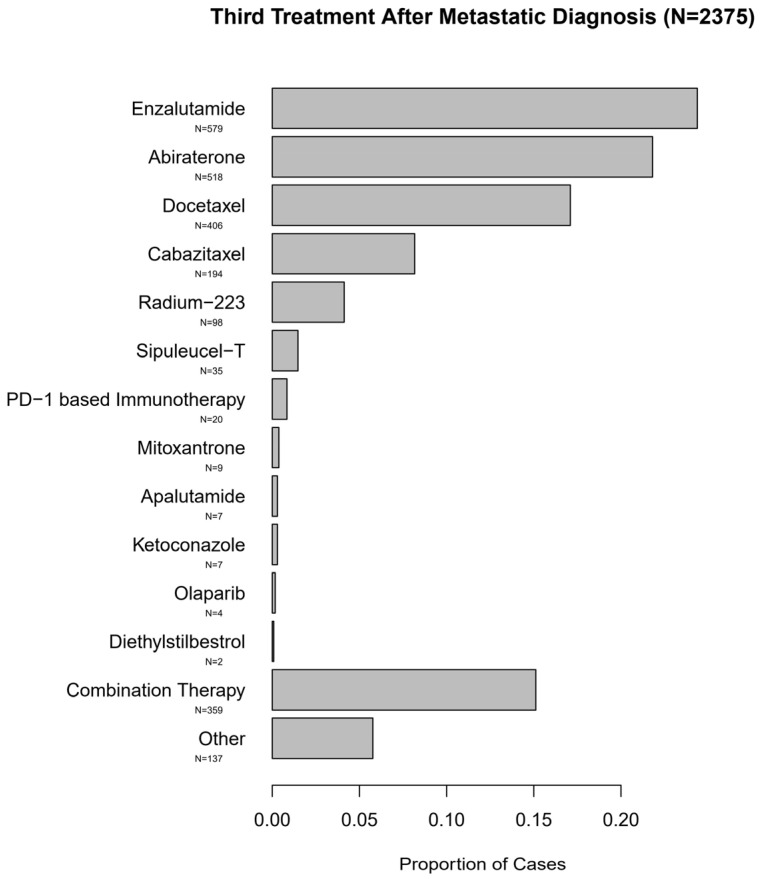
Third Treatment After Metastatic Diagnosis (*N* = 2375).

The predominant subsequent treatments in the entire cohort of these men with mPC consisted of NHTs, although other approved life-prolonging therapies were also utilized. These treatment patterns are summarized in Figure 4 and Figure 5.

### 3.2. Comparison of Effectiveness of NHT versus Docetaxel after a Prior NHT

Thereafter we aimed to evaluate the comparative effectiveness of alternate NHTs vs. docetaxel after treatment with an NHT in this real-world patient population. Out of the 9747 patients with mPC in the dataset, 1117 patients met all eligibility criteria for this analysis. The most common reason for exclusion were lack of information on any treatment other than ADT (*N* = 2733), 1L treatment other than abiraterone or enzalutamide (including *N* = 1588 1L docetaxel and *N* = 329 combination therapy), lack of information on 2L treatment (*N* = 2219) or 2L treatment other than abiraterone, enzalutamide, or docetaxel. Of these 1117 included patients, in the 1L therapy setting, 695 men received abiraterone, and 422 men received enzalutamide. In the 1L abiraterone group, 2L treatment consisted of enzalutamide in 508 and docetaxel in 187 patients. In the 1L enzalutamide group, 2L treatment consisted of abiraterone in 290 and docetaxel in 132 patients. Median follow-up for the study cohort was 9.8 months (range 0.1–64.4) and median follow-up among patients alive at the cutoff date for analysis was 12.5 months (range 0.2–64.4 months). 

Table S2 presents an extensive comparison of patient characteristics in alternate NHT and docetaxel groups in 2L. Propensity scores were estimated and used to calculate matching weights, and propensity score overlap (evaluated graphically Figure S1) and covariate balance (evaluated by standardized mean differences) were deemed satisfactory; more details are in the supplementary material. This suggests that analyses adjusted by using weighting based on the propensity score should largely eliminate potential confounding from the measured variables. 

### 3.3. Primary TTTTD and OS Analysis

**Figure 6:**
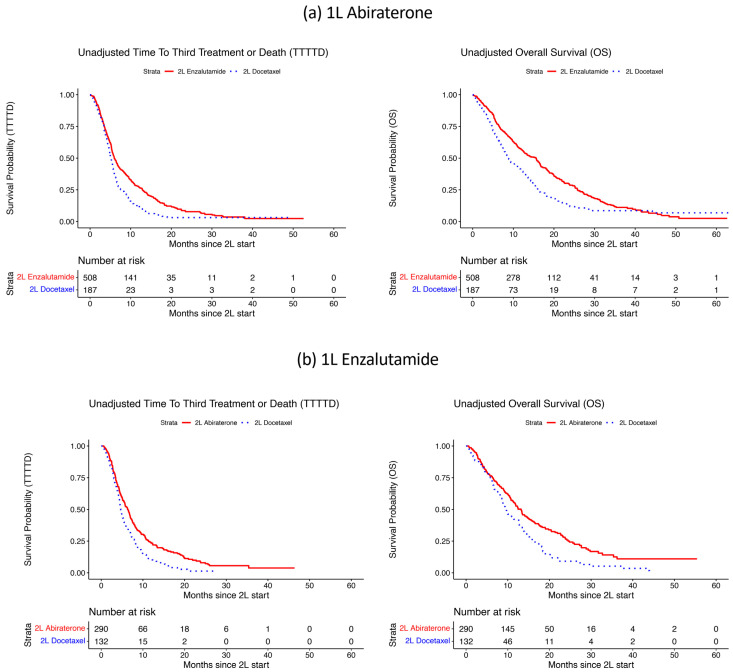
Unadjusted time to third treatment or death (TTTTD) and overall survival (OS) from 2L, with (**a**) 1L abiraterone (Figure 6a) and (**b**) 1L enzalutamide (Figure 6b).

Figure 6 displays Kaplan–Meier curves for the two survival outcomes of interest, TTTTD, and OS, for 2L NHT vs. docetaxel, separately for the 1L abiraterone (Figure 6a) and 1L enzalutamide (Figure 6b) patient groups. In both groups, 2L NHT showed evidence of superior survival experiences as compared with 2L docetaxel. Table 1 presents (unadjusted) median survival in all groups, as well as groups defined by time on 1L therapy (< or ≥6 months; < or ≥12 months). Median TTTTD was between 4.4 and 8.5 months across the 2L sub-groups and was longer in nearly all alternate NHT subgroups as compared with docetaxel. Median OS from the start of 2L therapy was consistently longer with alternate NHT as compared to docetaxel in both groups. In the 1L abiraterone group, the median OS with enzalutamide was 15.6 months as compared to 8.7 months with docetaxel. Similarly, in the 1L enzalutamide group, the median OS with abiraterone was 13.2 months as compared to 9.7 months with docetaxel.

Table 2 presents HRs from the Cox proportional hazards model adjusted using matching weights from the propensity score model for the overall population, and the results are consistent with the unadjusted results. The TTTTD HR for 2L docetaxel vs. alternate NHT was 1.26 (95% CI 1.04, 1.53) in the 1L abiraterone group and 1.32 (95% CI 1.07, 1.64) in the 1L enzalutamide group. The analogous HRs for OS were 1.36 (95% CI 1.09, 1.70) in the 1L abiraterone group and 1.40 (95% CI 1.09, 1.80) in the 1L enzalutamide group.

### 3.4. Subgroup Analyses

**Figure 7:**
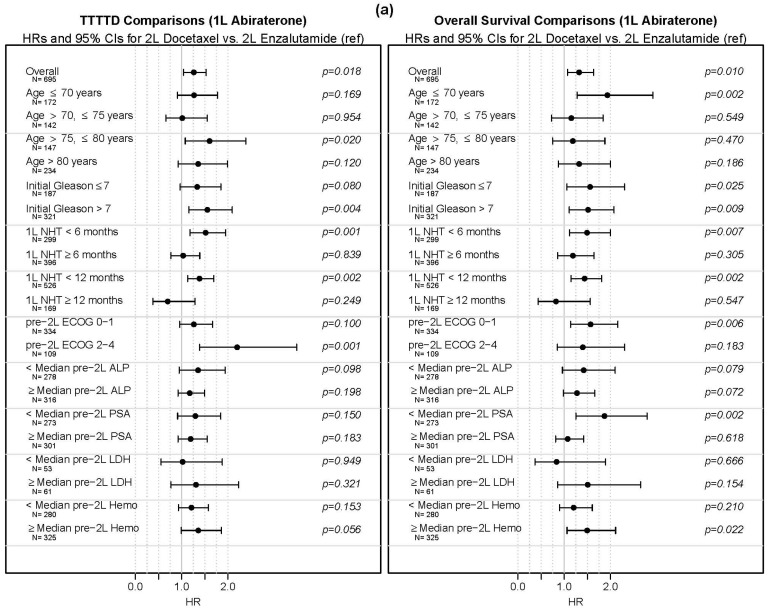

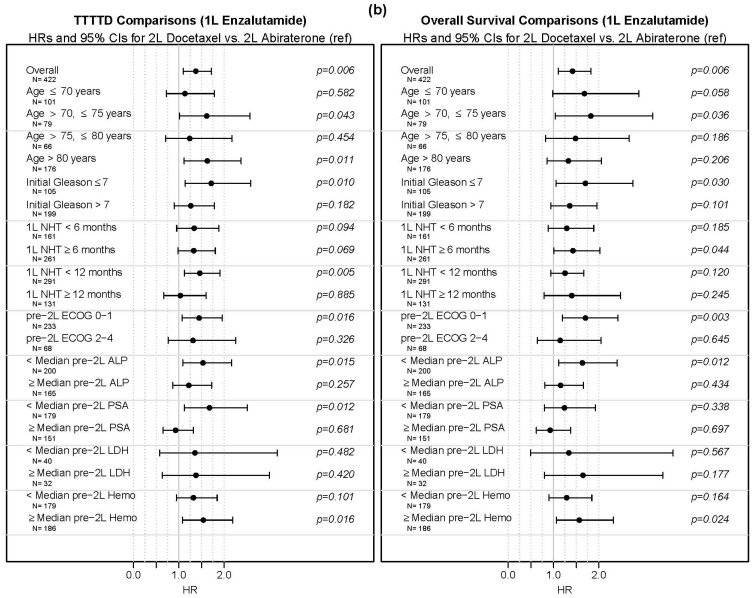
Time to third treatment (TTTTD) and overall survival (OS) comparisons between alternate novel hormonal therapy and docetaxel in patients treated with (**a**) first-line abiraterone (Figure 7a) and (**b**) first line-enzalutamide (Figure 7b).

In addition to the main analyses, subgroup analyses based on characteristics of interest were performed. Results from these are presented visually in Figure 7a,b and numerically in Table S3. With only a few exceptions, the HR point estimates for docetaxel vs. NHT were above 1 across subgroups and are not significantly less than 1 for any subgroup. Some subgroups with OS HRs significantly above 1 (at the 0.05 level) included age ≤ 70 (1L abiraterone); age 70–75 (1L enzalutamide); and ECOG 0–1 (1L abiraterone and 1L enzalutamide).

### 3.5. Post-2L Characteristics

Finally, we investigated selected post-2L characteristics, to evaluate whether any differences in these could help elucidate the main associations. Comparisons of these characteristics are presented in Table S4 and should be interpreted with some caution because the end of follow-up due to death or censoring could potentially impact the observed summaries. First, the median number of docetaxel cycles among patients in 2L docetaxel groups was 6 in both groups considered, which is lower than the recommended dose of docetaxel in men with mCRPC. Second, we evaluated ECOG after 4 months on 2L therapy, and before the end of 2L therapy; this was available for 35.8–40.0% patients across the arms. There was no evidence suggesting differences in this post-2L ECOG between 2L NHT and 2L docetaxel groups. 

The rates of 3L initiation were similar among the two arms in the 1L abiraterone group, but differed in the 1L enzalutamide group with 42.4% patients in the 2L abiraterone subgroup starting a 3L therapy as compared to 57.6% in the 2L docetaxel subgroup, despite a similar follow-up time between the groups. The total number of post-2L lines of treatment, the number of post-2L lines of treatment that include at least one of the approved life-extending drugs [6], and the number of unique post-2L life-extending drugs used [6] were similar among the 1L abiraterone patients, though nominally lower in the 2L docetaxel patients; these metrics were also similar among the 1L enzalutamide patients, though they tended to be nominally higher among 2L docetaxel patients.

## 4. Discussion

Once metastatic, prostate cancer is traditionally described either as mCSPC during which it can be treated by depleting the serum testosterone to castrate levels (<50 ng/dl), and mCRPC when prostate cancer continues to progress even in the presence of castrate levels of testosterone [12]. However, this difference is now losing its significance as the therapies which were utilized for mCRPC have been also approved for mCSPC. Upfront utilization of these therapies (NHT and docetaxel) has shown to improve OS and are recommended by current guidelines [6,12]. However, as discussed above, the real-world adaptation of these life-prolonging therapies in clinical practice, or the survival outcomes with docetaxel versus a NHT after treatment with one NHT are not well characterized. There has not been a randomized controlled trial in this setting either. 

There are four main contributions of this paper. First is the data on treatment patterns and utilization of systemic therapies in the real-world men with mPC in the United States. Until now, there are only a few published studies that have investigated the treatment patterns and use of upfront intensification in a real-world population. Upfront intensification with NHTs or docetaxel is currently recommended as initial treatment for patients with mCSPC [6]. Upfront intensification not only prolongs life but does so without compromising quality-of-life as observed in randomized clinical trials [13]. However, retrospective studies (mostly as abstracts) from multiple different databases including Optum, Medicare and ConcertAI Oncology Dataset have shown a consistent underutilization of intensification ranging from <10% to up to 30% of mCSPC patients and even those with visceral disease and in those with insurance [14,15,16,17]. In our dataset, we confirm that upfront intensification was low but a gradual and encouraging trend towards increased intensification was observed over the last 5 years (Figure 3). 

Second, we observe that in a large real-world dataset, more than two-thirds of patients with mPC treated with an NHT subsequently received an alternate NHT, and <30% of patients received docetaxel as a subsequent therapy. This suggests that an alternate NHT is widely used and is the preferred therapy over docetaxel in this real-world population in the United States. 

Third, we provide estimates of TTTTD and OS after disease progression on one NHT. These data are not currently available from prospective datasets. In our view, these estimates may be useful in the counseling of patients and for clinical trial design. 

Fourth, with the caveat of a non-randomized comparison and the retrospective nature of the study, we observed that the alternate NHT, when compared to docetaxel, was associated with superior OS after treatment with a prior NHT in mPC setting. It should be noted that efficacy data comparing docetaxel to alternate NHT after treatment with one NHT either from a clinical trial or a retrospective experience are currently non-existent.

Abiraterone, enzalutamide, and docetaxel have been shown to improve survival in men with metastatic castration-resistant prostate cancer [18,19,20,21,22,23]. Abiraterone is an irreversible inhibitor of 17 α-hydroxylase/17, 20-lyase (cytochrome P450c17 [CYP17A1]), blocks intratumoral production of testosterone, and one of its metabolites, Δ^4^-abiraterone is a direct androgen receptor (AR) inhibitor AR [24,25,26,27]. Enzalutamide impairs androgen binding to the AR, AR nuclear translocation, DNA binding, and coactivator recruitment [28]. Docetaxel exerts its anticancer effect at least in part by impairing AR signaling by inhibiting its nuclear translocation by stabilizing microtubules [29]. Preclinical studies have shown cross-resistance between these agents despite apparently differing mechanisms of action [8,30,31,32]. 

In a prospective single-arm trial (*n* = 215) in men with progressive mCRPC on abiraterone, the median time to PSA progression and radiographic progression with enzalutamide was 5.7 months and 8.1 months, respectively [7]. In a prospective, randomized, cross-over trial (*n* = 212) of abiraterone versus enzalutamide followed by cross-over to enzalutamide or abiraterone in progressive mCRPC, the median time to PSA progression with enzalutamide after abiraterone was 3.5 months while with abiraterone after enzalutamide was 1.7 months. This trial did not report radiographic progression-free survival with 2L NHT [7,8]. While the efficacy of an alternate NHT has been tested after a prior NHT in mCRPC in the two above-mentioned prospective trials, such prospective evaluation of the efficacy of docetaxel after prior treatment on an NHT has not yet been reported. However, in a post hoc analysis of the COU-AA-302 trial, the median duration of docetaxel treatment was ~3 months in men who had prior disease progression on abiraterone which is similar to that seen in our study [9]. Multiple other studies in this setting have shown similar results and are summarized in Table S5. At present, no evidence exists based on a randomized trial to support the use of an alternate NHT over docetaxel or vice versa after prior disease progression on one NHT for mPC. Furthermore, the estimates for TTTTD and OS are not available with 2L NHT or docetaxel after a prior 1L NHT as these agents have never been tested in any prospective clinical trial. Recent data from the CARD trial, which was conducted in the 3L systemic therapy setting, demonstrated significant improvement in clinical outcomes with cabazitaxel as compared to an alternate NHT in men with mCRPC who had previously received docetaxel and experienced disease progression within 12 months of being on the treatment of an NHT [33]. However, these data may not apply to 2L systemic therapy setting, i.e., to the patients who only received treatment with only one NHT in 1L, and a 2L therapy selection needs to be made between docetaxel and an alternate NHT. This lack of evidence on the comparative efficacy of alternate NHT versus docetaxel and survival estimates with these agents in the 2L setting poses serious challenges to treating providers on treatment selection, prognostication, and patient counseling. It is unlikely that we will have these data available from a randomized trial of 2L NHT or docetaxel in the near future, though the results of this study suggest that such a trial is warranted.

To better explain the lower OS with docetaxel, we evaluated multiple hypotheses. We investigated whether patients in the real-world were receiving fewer cycles of docetaxel, whether receipt of docetaxel was leading to early deterioration of performance status, or if there were differences in 3L therapies. In this real-world population, 2L docetaxel patients were indeed receiving fewer than recommended cycles of docetaxel (medians 5.5–6 cycles in the 2 groups) as opposed to ~10 cycles generally recommended in the clinical trial setting based on the results from the seminal TAX327 study which led to the approval of docetaxel in the mCRPC setting [23]. The proportions of patients initiating 3L treatment in the docetaxel and alternate NHT arms were similar in the 1L abiraterone group but greater for docetaxel vs. alternate NHT in the 1L enzalutamide group (Table S4). Similarly, the metrics comparing the number of life-extending agents were similar between docetaxel and alternate NHT in the 1L abiraterone group, but greater for docetaxel than alternate NHT in the 1L enzalutamide group. These findings may be a reflection of an earlier switch to subsequent therapy in those receiving 2L docetaxel possibly due to poor tolerability or earlier disease progression.

The strengths of our study include real-world patient-level data from a large cohort of patients with the propensity-weighted matching of multiple potential confounders. We report both TTTTD and OS with 2L docetaxel or alternate NHT, along with comprehensive subgroup analyses. 

The central weakness of this study is that our data are observational, and associations in observational data may be impacted by confounding. A priori, we would expect that physicians were selecting docetaxel for men whose cancer appeared to be more severe, in which case we would expect unadjusted estimates of TTTTD and OS to be shorter in that group, which we do indeed observe. If docetaxel was superior to alternate NHT after treatment with one NHT, we would expect that docetaxel would show similar or better performance than alternate NHT in the analyses where we adjust for measures of disease severity and treatment applicability; however, we did not observe this. Our approach to adjustment is designed to essentially match men based on key covariates that capture disease severity and the rapidity of progression, such as PSA, time on ADT, time on the first NHT, and key comorbidities. We did indeed see some indication of an imbalance in covariates between the treatment arms on some of these variables, but when using matching weights based on the propensity scores, the treatment arms became balanced, suggesting that confounding by the variables we have measured does not have a major impact on our results. However, our results may be impacted by confounding by variables we do not have measured, such as patient treatment preferences, healthcare access information, or genetic phenotypes that are not available to us. They may also be impacted by missingness, particularly if that missingness is differential—e.g., if patients in one treatment arm are typically missing PSA values only if they are very high, and that is not true of patients in the other treatment arm. Although these concerns are significant and urge caution in relying too strongly on results from this observational study, if docetaxel is in fact superior to alternate NHT in all men, it would require fairly strong effects of the unmeasured variables on both treatment selection and outcomes. Our results showing improvement in survival outcomes in the alternate NHT arms vs. docetaxel were very consistent across adjusted analyses and within subgroups. We therefore think it is worthwhile to share the results from this observational study, and strongly advocate for further studies investigating these treatment regimens to understand whether alternate NHT may be a better alternative than docetaxel for some patients. If a randomized clinical trial is unlikely, further observational studies with more careful annotation of potential confounders could better elucidate which treatment plan is superior and for which patients.

## 5. Conclusions

In this large observational real-world study, most men with new mPC did not receive NHT or docetaxel despite large, randomized trials showing significantly improved survival outcomes with these agents. The next step needs to be understanding the reasons for underutilization including lack of patient and physician awareness, barriers to access to these life-prolonging therapies including insurance/cost/access, fear of toxicities (drug or financial), or other reasons including co-morbidities, age, social, demographic or racial disparities. Once the causes are identified a combined and cohesive effort can be undertaken by various stakeholders to resolve them. In patients with mPC with prior treatment with only one NHT, the use of alternate NHT was more common and associated with superior survival compared to docetaxel in this retrospective dataset, which warrants a randomized controlled trial in this setting. These data also show survival estimates on NHT and docetaxel after progression on one NHT which may assist with designing clinical trials in this setting, as well as counseling and prognostication in the clinic. 

## Figures and Tables

**Table 1:** Median TTTTD and OS times, starting from the initiation of second-line (2L) therapy, overall and in subgroups defined by time on first-line (1L) therapy. Median survival estimates and 95% confidence intervals (CIs) are estimated using the Kaplan–Meier method, and are not adjusted for any covariates. Confidence limits that could not be estimated due to limited sample size are denoted by “-”. TTTTD is time to third-line treatment or death, and OS is overall survival.

1L Therapy	Population	Outcome	2L Therapy	*N*	Events	Median Survival, Months (95% CI)
Abiraterone	Overall	TTTTD	Enzalutamide	508	422	5.9 (5.6, 6.7)
			Docetaxel	187	161	5.1 (4.7, 5.7)
		OS	Enzalutamide	508	352	15.6 (12.7, 16.7)
			Docetaxel	187	144	8.7 (7.7, 11.6)
	1L NHT < 6 months	TTTTD	Enzalutamide	184	154	5.9 (5.4, 7.1)
			Docetaxel	115	104	5.0 (4.7, 5.7)
		OS	Enzalutamide	184	128	15.1 (11.0, 18.4)
			Docetaxel	115	91	9.1 (7.7, 12.1)
	1L NHT ≥ 6 months	TTTTD	Enzalutamide	324	268	6.0 (5.5, 7.0)
			Docetaxel	72	57	5.5 (4.3, 6.6)
		OS	Enzalutamide	324	224	15.6 (12.8, 16.7)
			Docetaxel	72	53	8.4 (6.3, 13.2)
	1L NHT < 12 months	TTTTD	Enzalutamide	362	306	5.6 (5.3, 6.4)
			Docetaxel	164	148	5.0 (4.6, 5.5)
		OS	Enzalutamide	362	263	12.5 (10.9, 15.6)
			Docetaxel	164	132	8.1 (7.3, 10.3)
	1L NHT ≥ 12 months	TTTTD	Enzalutamide	146	116	8.2 (5.9, 10.1)
			Docetaxel	23	13	8.5 (4.7, -)
		OS	Enzalutamide	146	89	18.9 (16.5, 24.6)
			Docetaxel	23	12	16.0 (12.4, -)
Enzalutamide	Overall	TTTTD	Abiraterone	290	226	6.3 (5.5, 7.0)
			Docetaxel	132	117	4.7 (4.4, 5.4)
		OS	Abiraterone	290	182	13.2 (11.4, 15.0)
			Docetaxel	132	103	9.7 (8.6, 12.6)
	1L NHT < 6 months	TTTTD	Abiraterone	92	72	4.8 (4.2, 6.3)
			Docetaxel	69	63	4.4 (3.7, 5.3)
		OS	Abiraterone	92	66	9.8 (6.7, 13.5)
			Docetaxel	69	58	7.0 (6.3, 9.3)
	1L NHT ≥ 6 months	TTTTD	Abiraterone	198	154	6.8 (6.2, 8.0)
			Docetaxel	63	54	5.3 (4.4, 8.2)
		OS	Abiraterone	198	116	14.0 (11.9, 17.6)
			Docetaxel	63	45	12.8 (10.9, 16.6)
	1L NHT < 12 months	TTTTD	Abiraterone	184	140	5.7 (5.0, 7.0)
			Docetaxel	107	96	4.5 (4.1, 5.3)
		OS	Abiraterone	184	125	10.4 (8.4, 12.0)
			Docetaxel	107	87	8.8 (7.7, 11.5)
	1L NHT ≥ 12 months	TTTTD	Abiraterone	106	86	6.9 (5.9, 8.5)
			Docetaxel	25	21	7.2 (4.9, 14.8)
		OS	Abiraterone	106	57	19.1 (14.7, 24.2)
			Docetaxel	25	16	15.1 (10.0, -)

**Table 2:** Hazard ratios (HRs) and 95% confidence intervals (CIs) comparing survival outcomes on second-line (2L) docetaxel vs. 2L alternate NHT. The HRs are from Cox proportional hazards models weighted using matching weights from propensity scores to adjust for potential confounding.

1L Therapy	Outcome	2L Therapy	*N*	HR (95% CI)	*p*
Abiraterone	TTTTD	Enzalutamide	508	1.00 (ref)	
		Docetaxel	187	1.26 (1.04, 1.53)	0.018
	OS	Enzalutamide	508	1.00 (ref)	
		Docetaxel	187	1.32 (1.07, 1.64)	0.009
Enzalutamide	TTTTD	Abiraterone	290	1.00 (ref)	
		Docetaxel	132	1.36 (1.09, 1.70)	0.008
	OS	Abiraterone	290	1.00 (ref)	
		Docetaxel	132	1.40 (1.09, 1.80)	0.009

## Data Availability

The data that support the findings of this study have been originated by Flatiron Health, Inc. These de-identified data may be made available upon request, and are subject to a license agreement with Flatiron Health; interested researchers should contact DataAccess@flatiron.com to determine licensing terms.

## Supplementary Materials

The following are available online at www.mdpi.com/article/10.3390/cancers13194951/s1, Table S1: Patient characteristics among all patients with evidence of contact within 180 days of metastatic prostate cancer diagnosis and with information on treatment in the metastatic setting, Table S2: Patient Characteristics Among 2L Alternate NHT vs. 2L Docetaxel, separately by 1L Abiraterone and 1L Enzalutamide, Table S3: Subgroup analyses comparing 2L Docetaxel vs. 2L NHT, separately by 1L Abiraterone and 1L Enzalutamide, Table S4: Comparison of patient characteristics after initiation of second line (2L) treatment, Table S5: Selected studies evaluating effectiveness of alternate novel hormonal therapies (NHT; abiraterone (A) or enzalutamide (E)) or docetaxel (D) after progression on an NHT (E or A), Figure S1: Smoothed histograms of propensity scores. Propensity scores model the probability of treatment with 2L Docetaxel vs. 2L Alternate NHT, and are presented for both 2L groups for the 1L Abiraterone patients (Supplementary Figure S1a) and 1L Enzalutamide patients (Supplementary Figure S1b).
