# Supplementary material for: Treatment Pattern and Outcomes with Systemic Therapy in Men with Metastatic Prostate Cancer in the Real-World Patients in the United States
Source: Cancers (Basel). 2021 Sep 30;13(19):4951. doi: 10.3390/cancers13194951 (PMC8508241; doi:10.3390/cancers13194951)

Supplementary Materials

Supplementary Table 1. Patient characteristics among all patients with evidence of contact within 180 days of metastatic prostate cancer diagnosis and with information on treatment in the metastatic setting.

Characteristic	N (%)
N	9747
Race / Ethnicity	
White (Non-Hispanic)	6242 (64%)
Black (Non-Hispanic)	881 (9%)
Asian (Non-Hispanic)	129 (1.3%)
Hispanic / Latino	626 (6.4%)
Other	1032 (10.6%)
Missing	837 (8.6%)
Gleason Score at Initial Diagnosis	
Less than or equal to 6	617 (6.3%)
3 + 4 = 7	656 (6.7%)
7 (when breakdown not available)	226 (2.3%)
4 + 3 = 7	828 (8.5%)
8	1497 (15.4%)
9	2441 (25%)
10	592 (6.1%)
Missing	2890 (29.7%)
PSA at Diagnosis of Metastasis - Median [Q1, Q3]	49.8 [12.5, 218.2]
Age at First Treatment After Metastatic Diagnosis - Median [Q1, Q3]	74.2 [66.6, 79.9]
Year of First Treatment After Metastatic Diagnosis*	
2011	1 (0%)
2012	15 (0.2%)
2013	1105 (11.3%)
2014	1416 (14.5%)
2015	1516 (15.6%)
2016	1610 (16.5%)
2017	1506 (15.5%)
2018	1514 (15.5%)
2019	1064 (10.9%)

***** In some cases, a treatment was given continuously both before and after metastatic diagnosis, in which case the start date reflects the true start date of the treatment, which may be prior to metastasis.

Supplementary Table 2. Patient Characteristics Among 2L Alternate NHT vs. 2L Docetaxel, separately by 1L Abiraterone and 1L Enzalutamide. Continuous variables are compared with the Wilcoxon rank-sum test, and categorical variables are compared with the chi-squared test. Columns display the p-values for these tests using both the original data and the propensity score (PS)-weighted data, as well as the standardized mean difference (SMD) using both the original data and the PS-weighted data. PS-weighted p-values below 0.05 and PS-weighted SMDs above 0.20 (a typical threshold used to identify lack of balance in treatment arms) are bold-faced. More details of evaluation of balance in treatment arms after PS-weighting is presented below.

	1L Abiraterone						1L Enzalutamide					
Variable	2L Enzalutamide	2L Docetaxel	p (unweighted)	p (PS-weighted)	SMD (unweighted)	SMD (PS-weighted)	2L Abiraterone	2L Docetaxel	p (unweighted)	p (PS-weighted)	SMD (unweighted)	SMD (PS-weighted)
N	508	187					290	132				
Follow-Up time (Months; Median [Q1, Q3])	10.2 [5.3, 17.2]						9.2 [4.9, 15.2]					
Follow-Up time Among Patients Alive Who Did Not Die On-Study (Months; Median [Q1, Q3])	13.6 [8.1, 19.4]						10.5 [6.4, 17.6]					
Age at 2L Start (Years; Median [Q1, Q3])	77.9 [70.9, 81.3]	74.2 [67.0, 79.2]	<0.001	**0.032**	0.394	0.192	79.9 [71.9, 82.2]	73.4 [69.2, 79.7]	<0.001	0.058	0.408	0.166
Time On 1L Therapy (Months; Median [Q1, Q3])	7.7 [4.6, 13.1]	5.1 [3.0, 8.3]	<0.001	**0.028**	0.466	0.168	8.9 [5.3, 15.4]	5.5 [3.3, 10.9]	<0.001	0.072	0.387	0.168
Hormone Sensitive in 1L	4.3%	3.7%	0.897	0.690	0.030	0.036	3.8%	2.3%	0.606	0.416	0.089	0.093
Year at 2L Start												
2013	8 (1.6%)	11 (5.9%)	0.013	0.637	0.228	0.115	2 (0.7%)	3 (2.3%)	0.098	0.589	0.131	0.064
2014	65 (12.8%)	29 (15.5%)			0.078	0.015	9 (3.1%)	4 (3.0%)			0.004	0.060
2015	98 (19.3%)	30 (16.0%)			0.085	0.036	37 (12.8%)	20 (15.2%)			0.069	0.039
2016	112 (22.0%)	29 (15.5%)			0.168	0.108	78 (26.9%)	21 (15.9%)			0.270	0.173
2017	88 (17.3%)	28 (15.0%)			0.064	0.030	64 (22.1%)	30 (22.7%)			0.016	<0.001
2018	120 (23.6%)	50 (26.7%)			0.072	0.063	80 (27.6%)	37 (28.0%)			0.010	0.025
2019	17 (3.3%)	10 (5.3%)			0.098	0.095	20 (6.9%)	17 (12.9%)			0.201	0.153
ECOG Prior to 2L Start												
0	88 (17.3%)	36 (19.3%)	0.593	0.310	0.050	0.066	58 (20.0%)	27 (20.5%)	0.107	**0.039**	0.011	0.023
1	151 (29.7%)	59 (31.6%)			0.040	0.084	103 (35.5%)	45 (34.1%)			0.030	0.017
2	67 (13.2%)	18 (9.6%)			0.112	0.157	29 (10.0%)	21 (15.9%)			0.176	0.173
3-4	17 (3.3%)	7 (3.7%)			0.021	0.015	16 (5.5%)	2 (1.5%)			0.218	**0.292**
Missing	185 (36.4%)	67 (35.8%)			0.012	0.031	84 (29.0%)	37 (28.0%)			0.021	0.002
Race / Ethnicity												
White (Non-Hispanic)	335 (65.9%)	127 (67.9%)	0.779	0.562	0.042	0.091	191 (65.9%)	97 (73.5%)	0.361	0.589	0.166	0.133
Black (Non-Hispanic)	52 (10.2%)	19 (10.2%)			0.002	0.064	33 (11.4%)	13 (9.8%)			0.050	0.089
Asian (Non-Hispanic)	8 (1.6%)	1 (0.5%)			0.102	0.111	3 (1.0%)	2 (1.5%)			0.043	0.096
Hispanic / Latino	41 (8.1%)	12 (6.4%)			0.064	0.077	13 (4.5%)	6 (4.5%)			0.003	0.003
Other	46 (9.1%)	18 (9.6%)			0.020	0.043	35 (12.1%)	8 (6.1%)			0.210	0.107
Missing	26 (5.1%)	10 (5.3%)			0.010	0.015	15 (5.2%)	6 (4.5%)			0.029	0.059
Gleason At Initial Diagnosis												
Less than or equal to 6	38 (7.5%)	14 (7.5%)	0.271	0.594	<0.001	0.020	24 (8.3%)	10 (7.6%)	0.817	0.977	0.026	0.040
3 + 4 = 7	35 (6.9%)	12 (6.4%)			0.019	0.010	17 (5.9%)	10 (7.6%)			0.068	0.063
7 (when breakdown not available)	24 (4.7%)	6 (3.2%)			0.078	0.035	10 (3.4%)	5 (3.8%)			0.018	0.038
4 + 3 = 7	36 (7.1%)	22 (11.8%)			0.160	0.142	18 (6.2%)	11 (8.3%)			0.082	0.058
8	89 (17.5%)	25 (13.4%)			0.115	0.084	45 (15.5%)	17 (12.9%)			0.075	0.076
9	114 (22.4%)	50 (26.7%)			0.100	0.038	73 (25.2%)	41 (31.1%)			0.131	0.042
10	34 (6.7%)	9 (4.8%)			0.081	0.111	13 (4.5%)	10 (7.6%)			0.130	0.050
Missing	138 (27.2%)	49 (26.2%)			0.022	0.017	90 (31.0%)	28 (21.2%)			0.224	0.125
PSA at Diagnosis of Metastasis	46.0 [13.9, 150.0]	53.6 [13.6, 239.0]	0.455	0.447	0.060	0.046	48.8 [12.8, 165.8]	50.4 [9.3, 238.1]	0.887	0.332	0.034	0.102
Missing (%)	11.2%	7.5%	0.194	0.199			11.7%	13.6%	0.693	0.981		
PSA at within 3 Months Prior to 1L Start	28.6 [10.6, 86.2]	48.3 [14.0, 102.6]	0.033	0.870	0.112	0.109	25.2 [8.5, 85.4]	46.2 [21.9, 137.7]	0.002	0.198	0.098	0.010
Missing (%)	37.8%	35.8%	0.699	0.994			36.6%	43.2%	0.234	0.468		
Biomarkers within 3 Months Prior to 2L Start												
PSA	32.7 [9.0, 103.3]	74.3 [25.3, 266.9]	<0.001	0.108	0.107	0.105	24.8 [8.6, 74.7]	66.9 [19.3, 187.0]	<0.001	0.064	0.354	0.166
Missing (%)	19.3%	12.3%	0.041	0.466			22.1%	21.2%	0.944	0.765		
LDH	207.0 [170.0, 269.0]	265.0 [201.0, 335.5]	0.016	0.478	0.124	0.056	205.0 [180.0, 290.0]	201.0 [173.0, 467.5]	0.570	0.777	0.083	0.017
Missing (%)	83.7%	83.4%	1.000	0.717			83.1%	82.6%	1.000	0.977		
Alkaline Phosphatase	90.0 [65.0, 150.5]	168.0 [83.5, 340.5]	<0.001	0.058	0.281	0.011	86.0 [67.0, 123.0]	103.0 [77.0, 162.0]	0.001	0.258	0.200	0.018
Missing (%)	16.7%	8.6%	0.010	0.470			14.5%	11.4%	0.474	0.923		
Hemoglobin	12.2 [11.1, 13.1]	11.6 [10.4, 13.1]	0.010	0.668	0.202	0.025	12.2 [11.1, 13.3]	11.9 [10.6, 13.2]	0.136	0.635	0.260	0.144
Missing (%)	14.8%	8.0%	0.026	0.753			16.2%	7.6%	0.024	0.186		
Total Number if Diagnosis Codes	6.0 [4.0, 11.0]	7.0 [5.0, 12.0]	0.006	0.140	0.145	0.092	7.0 [3.0, 11.8]	8.0 [5.0, 14.0]	0.029	0.171	0.002	0.034
Diagnoses Noted in Medical Records Prior to 2L Start												
Any Specific Mets Noted	71.5%	77.5%	0.132	0.290	0.140	0.096	70.3%	74.2%	0.480	0.570	0.087	0.063
Visceral Mets	1.6%	3.7%	0.147	0.318	0.135	0.092	0.3%	2.3%	0.176	0.375	0.170	0.110

Diabetes	9.8%	7.5%	0.421	0.191	0.084	0.125	12.8%	8.3%	0.245	0.252	0.144	0.130
Heart Failure	2.0%	1.1%	0.632	0.289	0.073	0.104	1.7%	0.8%	0.738	0.522	0.087	0.076
Neuropathy	2.4%	1.1%	0.441	0.198	0.100	0.126	1.7%	0.8%	0.738	0.171	0.087	0.142
Insurance At/Prior to Second Line Start (% Reporting)												
Commercial Insurance	33.3%	33.2%	1.000	0.623	0.002	0.045	42.1%	37.1%	0.394	0.743	0.101	0.036
Medicaid	2.0%	2.1%	1.000	0.350	0.012	0.093	3.8%	1.5%	0.341	0.175	0.142	0.162
Medicare	22.2%	21.4%	0.891	0.668	0.021	0.038	24.1%	23.5%	0.982	0.928	0.015	0.010
Other Government Program	3.7%	2.7%	0.654	0.951	0.060	0.005	4.8%	5.3%	1.000	0.981	0.022	0.003
Other Payer	28.5%	20.9%	0.052	0.280	0.179	0.099	19.0%	26.5%	0.104	0.226	0.180	0.133
Patient Assistance Program	7.9%	11.2%	0.217	0.308	0.114	0.089	4.1%	7.6%	0.216	0.636	0.146	0.055
Missing	13.4%	18.2%	0.143	0.250	0.132	0.101	12.8%	9.8%	0.487	0.386	0.092	0.098

Supplementary Table 3. Subgroup analyses comparing 2L Docetaxel vs. 2L NHT, separately by 1L Abiraterone and 1L Enzalutamide. The sample size and proportion of the total study size are reported; some proportions add to less than 100% when the variable has missing values. Hazard Ratios (HRs) HRs, 95% confidence intervals, and p-values are presented from Cox proportional hazards models adjusted for confounding via propensity-score weighting.

	1L Abiraterone (HRs for 2L Docetaxel vs. 2L Enzalutamide)					1L Enzalutamide (HRs for 2L Docetaxel vs. 2L Abiraterone)				
Subgroup	N	HR (TTTTD)	p (TTTTD)	HR (OS)	p (OS)	N	HR (TTTTD)	p (TTTTD)	HR (OS)	p (OS)
Overall	695 (100.0%)	1.26 (1.04, 1.53)	0.018	1.32 (1.07, 1.63)	0.010	422 (100.0%)	1.37 (1.10, 1.72)	0.006	1.42 (1.10, 1.82)	0.006
Age ≤ 70 years	172 (24.7%)	1.27 (0.90, 1.78)	0.169	1.93 (1.28, 2.91)	0.002	101 (23.9%)	1.13 (0.72, 1.78)	0.582	1.68 (0.98, 2.88)	0.058
Age > 70 years and ≤ 75 years	142 (20.4%)	1.01 (0.66, 1.55)	0.954	1.15 (0.72, 1.84)	0.549	79 (18.7%)	1.61 (1.01, 2.57)	0.043	1.82 (1.04, 3.19)	0.036
Age > 75 years and ≤ 80 years	147 (21.2%)	1.60 (1.08, 2.39)	0.020	1.19 (0.75, 1.88)	0.470	66 (15.6%)	1.24 (0.71, 2.17)	0.454	1.49 (0.83, 2.67)	0.186
Age > 80 years	234 (33.7%)	1.36 (0.92, 1.99)	0.120	1.32 (0.87, 2.00)	0.186	176 (41.7%)	1.63 (1.12, 2.37)	0.011	1.33 (0.86, 2.05)	0.206
Initial Gleason ≤ 7	187 (26.9%)	1.34 (0.97, 1.85)	0.080	1.56 (1.06, 2.30)	0.025	105 (24.9%)	1.72 (1.14, 2.58)	0.010	1.70 (1.05, 2.75)	0.030
Initial Gleason > 7	321 (46.2%)	1.55 (1.16, 2.09)	0.004	1.52 (1.11, 2.07)	0.009	199 (47.2%)	1.26 (0.90, 1.78)	0.182	1.36 (0.94, 1.96)	0.101
1L NHT < 6 months	299 (43.0%)	1.52 (1.18, 1.95)	0.001	1.49 (1.12, 1.99)	0.007	161 (38.2%)	1.34 (0.95, 1.88)	0.094	1.29 (0.88, 1.89)	0.185
1L NHT ≥ 6 months	396 (57.0%)	1.03 (0.77, 1.39)	0.839	1.19 (0.86, 1.64)	0.305	261 (61.8%)	1.33 (0.98, 1.80)	0.069	1.43 (1.01, 2.02)	0.044
1L NHT < 12 months	526 (75.7%)	1.38 (1.13, 1.69)	0.002	1.44 (1.14, 1.81)	0.002	291 (69.0%)	1.46 (1.12, 1.91)	0.005	1.25 (0.94, 1.67)	0.120
1L NHT ≥ 12 months	169 (24.3%)	0.70 (0.38, 1.29)	0.249	0.82 (0.43, 1.56)	0.547	131 (31.0%)	1.03 (0.67, 1.60)	0.885	1.40 (0.79, 2.48)	0.245
pre-2L ECOG 0-1	334 (48.1%)	1.26 (0.96, 1.67)	0.100	1.57 (1.14, 2.16)	0.006	233 (55.2%)	1.45 (1.07, 1.96)	0.016	1.70 (1.20, 2.42)	0.003
pre-2L ECOG 2-4	109 (15.7%)	2.20 (1.39, 3.48)	0.001	1.40 (0.85, 2.30)	0.183	68 (16.1%)	1.31 (0.76, 2.25)	0.326	1.15 (0.64, 2.05)	0.645
< Median pre-2L ALP	278 (40.0%)	1.35 (0.95, 1.94)	0.098	1.42 (0.96, 2.10)	0.079	200 (47.4%)	1.53 (1.09, 2.16)	0.015	1.64 (1.12, 2.40)	0.012
≥ Median pre-2L ALP	316 (45.5%)	1.17 (0.92, 1.50)	0.198	1.27 (0.98, 1.66)	0.072	165 (39.1%)	1.22 (0.86, 1.72)	0.257	1.16 (0.80, 1.66)	0.434
Below Median pre-2L PSA	273 (39.3%)	1.30 (0.91, 1.84)	0.150	1.87 (1.25, 2.79)	0.002	179 (42.4%)	1.68 (1.12, 2.51)	0.012	1.24 (0.80, 1.92)	0.338
≥ Median pre-2L PSA	301 (43.3%)	1.19 (0.92, 1.55)	0.183	1.07 (0.81, 1.42)	0.618	151 (35.8%)	0.93 (0.65, 1.32)	0.681	0.92 (0.62, 1.38)	0.697
Below Median pre-2L LDH	53 (7.6%)	1.02 (0.55, 1.88)	0.949	0.83 (0.37, 1.89)	0.666	40 (9.5%)	1.36 (0.58, 3.17)	0.482	1.34 (0.49, 3.64)	0.567
≥ Median pre-2L LDH	61 (8.8%)	1.31 (0.77, 2.23)	0.321	1.51 (0.86, 2.65)	0.154	32 (7.6%)	1.38 (0.63, 3.00)	0.420	1.65 (0.80, 3.41)	0.177
Below Median pre-2L Hemo	280 (40.3%)	1.21 (0.93, 1.57)	0.153	1.20 (0.90, 1.61)	0.210	179 (42.4%)	1.32 (0.95, 1.85)	0.101	1.29 (0.90, 1.85)	0.164
≥ Median pre-2L Hemo	325 (46.8%)	1.36 (0.99, 1.86)	0.056	1.50 (1.06, 2.11)	0.022	186 (44.1%)	1.54 (1.08, 2.19)	0.016	1.57 (1.06, 2.32)	0.024

Supplementary Table 4. Comparison of patient characteristics after initiation of second line (2L) treatment. Presented are the number of docetaxel administrations among patients taking 2L docetaxel; ECOG measurements available at least 4 months after 2L start and before 2L end; and summary statistics of treatment in third line and beyond. Also presented are p-values from t-tests comparing the 2L treatment groups.

	1L Abiraterone			1L Enzalutamide		
Characteristic	2L Enzalutamide	2L Docetaxel	p	2L Abiraterone	2L Docetaxel	p
N	508	187		290	132	
Number of 2L Docetaxel Administrations (Median [Q1, Q3])		6 (3.5, 9)			6 (3, 8)	
ECOG (4 Months After 2L Start - End of 2L)						
N Available (%)	203 (40.0%)	67 (35.8%)		111 (38.3%)	50 (37.9%)	
Mean ± SD	1.15 ± 0.84	0.99 ± 0.75	0.161	1.07 ± 0.82	1.14 ± 0.86	0.631
Treatment Post-2L						
Any Third Line Therapy Observed	259 (51.0%)	93 (49.7%)		123 (42.4%)	76 (57.6%)	
Total Post-2L Lines of Therapy: Mean ± SD	0.91 ± 1.12	0.86 ± 1.14	0.605	0.76 ± 1.13	0.96 ± 1.16	0.095
Post-2L Lines of Therapy Including Any Approved Drug: Mean ± SD	0.81 ± 1.00	0.74 ± 0.98	0.415	0.67 ± 0.99	0.79 ± 0.87	0.236
Unique Post-2L Approve Drugs: Mean ± SD	0.85 ± 1.05	0.78 ± 1.02	0.421	0.70 ± 1.02	0.79 ± 0.86	0.407

Supplementary Table 5. Selected studies evaluating effectiveness of alternate novel hormonal therapies (NHT; abiraterone (A) or enzalutamide (E)) or docetaxel (D) after progression on an NHT (E or A)

Author	Sample size (n)	Patient population	PSA_{50} response rate (%)	Median PFS per PCWG2 criteria in months	Median OS from first NHT	PMID
NHT after progression on NHT						
Suzman et al	30	Enzalutamide after progression on abiraterone, docetaxel naive	34	4.7	Not reported	25053178
Maughan et al	65	Alternate NHT (A or E) after progression on NHT, prior docetaxel allowed	37 (A→E) and 13 (E→A)		33.3 (A→E) and 29.9 (E→A)	27527643
de Bono et al	214	E after progression on ≥24 weeks of A, prior docetaxel allowed	27	5.7	Not reached	28844372
Matsubara et al	97	Alternate NHT (A or E) after progression on NHT, chemo naïve	30 (A→E) and 6.4 (E→A)	3.4 (A→E) and 2.9 (E→A) months	25.4 (A→E) and 24.2 (E→A) months	29042308
Azad et al	115	E after A, prior docetaxel allowed (n=68)	23.5 (22% in prior docetaxel and 25% in docetaxel naïve)	5.3 (4.6 in prior docetaxel and 6.6 in docetaxel naïve)	10.6 (10.6 in prior docetaxel and 8.6 in docetaxel naïve)	28314611
Khalaf et al	202	101 (A→E) and 101 (E→A), prior docetaxel allowed	NR	2.7 (A→E) and 1.7 (E→A)	NR	31727538
Docetaxel after progression on NHT (abiraterone)						
Suzman et al	31	Docetaxel after progression on abiraterone	40	4.4	NR	25053178
de Bono et al	261		40 (100 patients with PSA values)	3 (Median duration)	NR	
Schweizer et al	24		38	4.4	NR	24491307
Azad et al	37		32	NR	NR	25175831
Mezynski et al	35		26	NR	12.5 months from start of docetaxel	22771826

Propensity Score Evaluation: Covariate balance was assessed by examining p-values for weighted versions of the tests in Supplementary Table 2 (Wilcoxon rank-sum tests and chi-squared tests), and by examining the weighted standardized mean differences (SMDs). Among 1L abiraterone patients, the standardized mean differences (SMD) were below 0.20 for all variables, a typical threshold used to indicate reasonable covariate balance. The variables with statistically significant differences even after PS-weighting were age at 2L start and time on 1L therapy; these were investigated in subgroup analyses. Among 1L enzalutamide patients, the only variable above the 0.20 threshold was the frequency of ECOG 3-4 patients; there were very few of these patients, so this likely does not impact our results, though the results should be interpreted cautiously when applied to such patients. Smoothed histograms of the propensity score distributions (Supplementary Figure 1) show they are highly overlapping. We also calculated the area under the receiver-operator characteristic curve (AUC) for evaluating how well the propensity score predicted the observed treatment; among 1L abiraterone patients, this AUC was 0.71 (95% CI 0.67-0.75); among 1L enzalutamide patients, the AUC was 0.68 (95% CI 0.63-0.74).

Supplementary Figure 1. Smoothed histograms of propensity scores. Propensity scores model the probability of treatment with 2L Docetaxel vs. 2L Alternate NHT, and are presented for both 2L groups for the 1L Abiraterone patients (Supplementary Figure 1a) and 1L Enzalutamide patients (Supplementary Figure 1b).

(a)

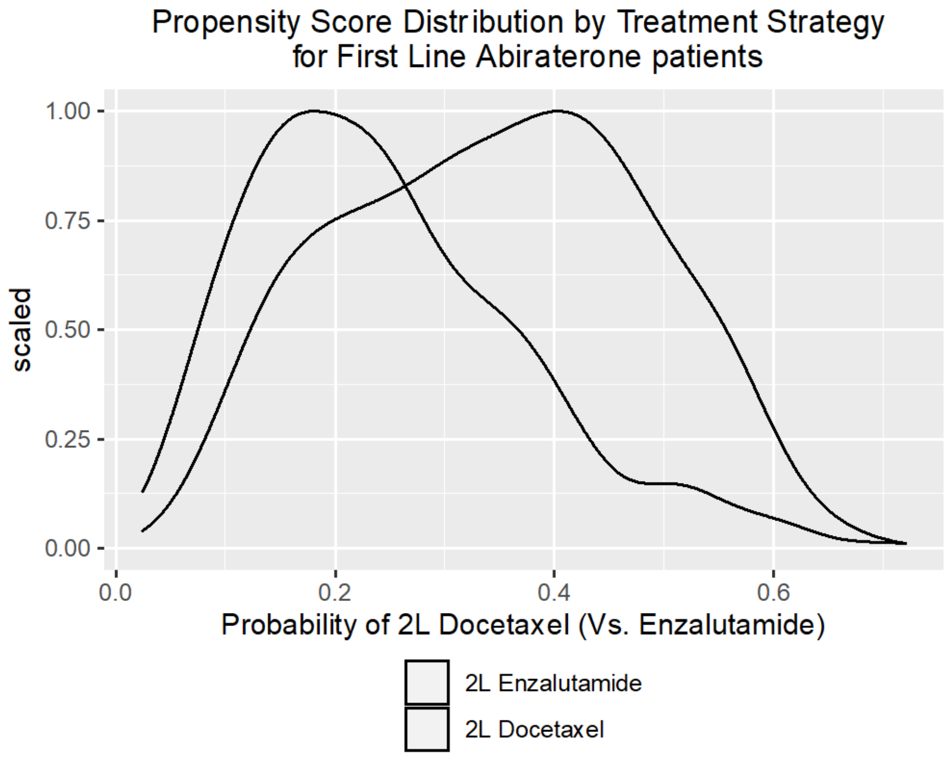

(b)

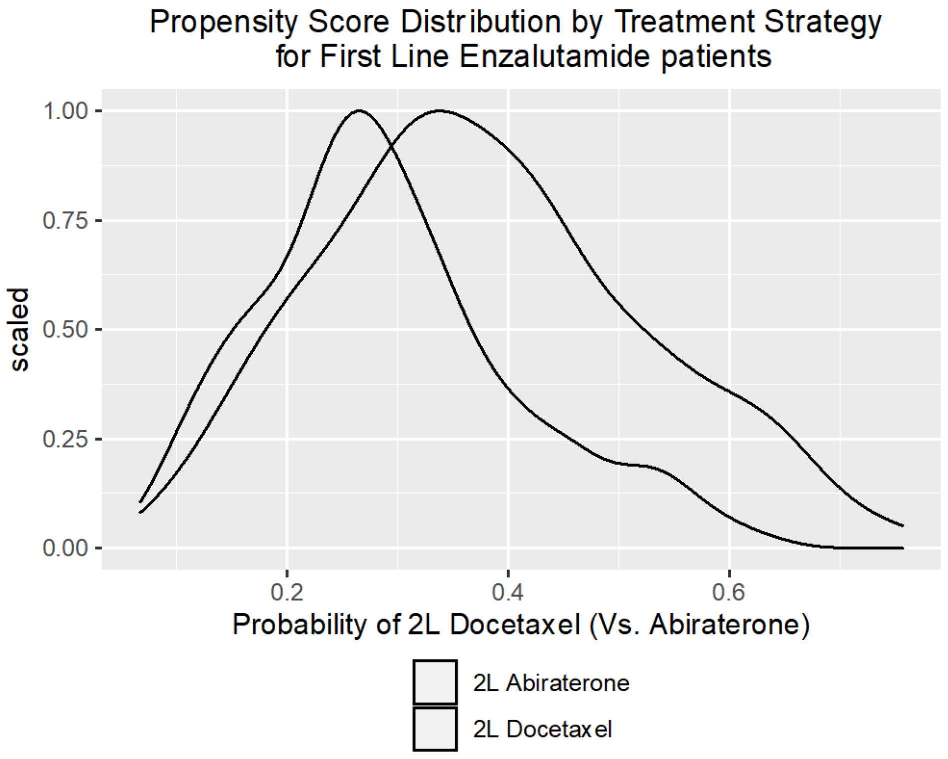

Supplement: Supplementary file 1 [file cancers-13-04951-s001.zip › cancers-1391330-supplementary.pdf]
